# Supplementary material for: End Sequence Analysis Toolkit (ESAT) expands the extractable information from single-cell RNA-seq data
Source: Genome Res. 2016 Oct;26(10):1397–410. doi: 10.1101/gr.207902.116 (PMC5052061; doi:10.1101/gr.207902.116)
Supplement: Supplemental Material [file supp_gr.207902.116_Supp_Materials.tar.gz › ESAT-esat_v0.1/doc/html/classumms_1_1esat_1_1_e_s_a_t_distance_calculator-members.html]

ESAT: Member List


ESAT


- Main Page
- Related Pages
- Packages
- Classes
- Files

- Class List
- Class Index
- Class Hierarchy
- Class Members

All Classes Namespaces Files Functions Variables Enumerator Pages

- umms
- esat
- ESATDistanceCalculator

umms.esat.ESATDistanceCalculator Member List

This is the complete list of members for umms.esat.ESATDistanceCalculator, including all inherited members.

|  |  |  |
| --- | --- | --- |
| array2List(double[] array) | umms.esat.ESATDistanceCalculator | static |
| calculateNormalizationFactors(MatrixWithHeaders mat) | umms.esat.ESATDistanceCalculator | static |
| ESATDistanceCalculator(String[] args) | umms.esat.ESATDistanceCalculator |  |
| getSubAnnotationFromEnd(Gene annotation, int length, int distanceFromTranscriptEnd) | umms.esat.ESATDistanceCalculator | static |
| getSubAnnotationFromStart(Gene annotation, int length, int distanceFromTranscriptStart) | umms.esat.ESATDistanceCalculator | static |
| main(String[] args) | umms.esat.ESATDistanceCalculator | static |
| reconstructGeneEnds(BEDFileParser completeReconstruction, BEDFileParser annotations, String annotationFile) | umms.esat.ESATDistanceCalculator | static |
| whitespaceDelimiter | umms.esat.ESATDistanceCalculator | static |
| writeNormalizedMatrix(String fileName, MatrixWithHeaders resultMatrix) | umms.esat.ESATDistanceCalculator | static |


---

Generated on Fri Aug 15 2014 15:11:15 for ESAT by  

 1.8.7
